# Maintaining Psychotropic Medication Adherence Among War-Affected Patients: Lessons from Gaza

**DOI:** 10.1192/bjo.2026.11183

**Published:** 2026-06-30

**Authors:** Ali Abunemer, Noor Al-Maharmah, Rawan Al-Habbash

**Affiliations:** Gaza Community Mental Health Programme, Gaza City, Palestine

## Abstract

**Aims:**

Background: Armed conflict severely disrupts the continuity of psychiatric care, particularly for patients dependent on long-term psychotropic medication. During the recent Gaza war, widespread displacement of patients and medical personnel and destruction of the health service infrastructure created unprecedented barriers to treatment continuity. Mental health professionals were compelled to develop innovative, context-specific solutions to prevent clinical deterioration and safeguard patient stability.

Aims were to identify the main challenges affecting psychotropic medication adherence among war-affected patients in Gaza and to describe adaptive strategies that supported treatment continuity under crisis conditions.

**Methods:**

This study was conducted between October 2023 and January 2024 within five community mental health centres and two psychiatric hospitals in Gaza. This was a mixed-methods study, that used triangulated data from semi-structured interviews with clinicians, focus group discussions with multi-disciplinary teams, clinical records and medication tracking data. Thematic analysis of interviews and focus groups was used to identify recurring patterns of non-adherence, contributing factors, and responsive interventions. Themes were identified and refined through iterative discussions to ensure they reflected clinical observations across sites. Descriptive statistics were used to describe reasons for medication interruption as recorded in clinical records.

**Results:**

Medication discontinuation was due to drug shortages (45%), forced displacement (33%), and restricted mobility caused by security risks (22%). Thematic analysis identified several adaptive strategies: decentralised medication distribution through NGO partnerships, telepsychiatry follow-ups, substitution with pharmacological equivalents, and family engagement in adherence monitoring. Clinicians reported that these strategies reduced relapse rates and preserved treatment stability in most high-risk cases.

**Conclusion:**

Maintaining psychotropic medication adherence during armed conflict is both a clinical and ethical imperative. The Gaza experience demonstrates that flexible, community-driven, and ethically grounded models of care can sustain treatment continuity in extreme humanitarian settings. Strengthening emergency medication supply chains and integrating psychosocial support into humanitarian response frameworks are essential to protect psychiatric outcomes in conflict-affected populations.

